# SARS-CoV-2/COVID-19 Vaccines: The Promises and the Challenges Ahead

**DOI:** 10.3390/vaccines9010021

**Published:** 2021-01-04

**Authors:** Vincenzo Baldo, Chiara Reno, Silvia Cocchio, Maria Pia Fantini

**Affiliations:** 1 Department of Cardiac, Thoracic, Vascular Sciences and Public Health, Hygiene and Public Health Unit, University of Padua, 35100 Padua, Italy; silvia.cocchio@unipd.it; 2 Department of Biomedical and Neuromotor Sciences, Alma Mater Studiorum—University of Bologna, 40126 Bologna, Italy; chiara.reno@studio.unibo.it (C.R.); mariapia.fantini@unibo.it (M.P.F.)

The development of a new vaccine usually consists of a linear sequence of several steps and lasts many years [1]. The sudden outbreak of the SARS-CoV-2/COVID-19 pandemic urged for a quick response and led to an unprecedented effort to produce a vaccine against this virus in a very short time, in the context of very limited pre-existing clinical experience with any coronavirus vaccine and with multiple steps of development carried out in parallel, with the deployment of different technologies, some of which have never been used in a licensed vaccine before [2,3]. There is a strong need for vaccines against SARS-CoV-2 as they potentially represent a strong tool to counteract the spread of the virus in combination with non-pharmaceutical interventions that, although essential, are not able to control it sustainably [4], and in the absence of an effective therapeutic protocol against COVID-19. Based on disease and immune response knowledge acquired over the course of the pandemic, vaccines should induce high affinity virus-neutralizing antibodies specific for SARS-CoV-2 to optimally prevent infection and unfavorable events like severe disease and death. Namely, these neutralizing antibodies should be directed against a particular domain (the so-called receptor binding domain—RBD) within the spike (S) protein, which interacts with target cell ACE2 receptors [2]. The primary immune mechanism of avoiding infection is through blocking viral attachment to target cells. Of note, S protein vaccination may induce un-wanted antibodies in addition to the neutralizing ones directed against the RBD. Therefore, it is important to construct a vaccine displaying the RBD—or even only the RIS (Receptor Interaction Site) conformation instead of the whole S protein—as other antibodies might sustain the risk of disease enhancement as demonstrated in the past for some inactivated coronavirus vaccines in animal models [2].

The herd immunity threshold for SARS-CoV-2 has been calculated to range between 50% and 67%, with the assumption of no population immunity, in the absence of any interventions and considering all individuals are equally susceptible and infectious [5]. Certainly, the durability of immune memory is key in sustaining herd immunity [5]. Seroprevalence studies carried out worldwide suggest that less of 10% of the included population has been infected, highlighting that the vast majority of individuals are still susceptible to the infection [6]. 

The different types of vaccines under development include inactivated or weakened virus (virus vaccines), protein subunit or virus-like particles (protein-based vaccines) and replicating or non-replicating viral vector vaccines. [3,7]. However, the real novelty is represented by nucleic acid vaccines (DNA- or RNA-based) that are designed to insert genetic instructions, mainly for virus spike’s pieces production, into the human cell; so far this kind of technology has not been used in approved vaccines [3]. This method shows great potential because no culture or fermentation is required, allowing for a fast production [1].

As of 23 December 2020, after more than eleven months since the isolation of the strain of the new coronavirus, there are 287 vaccines candidates, of which 224 are at the pre-clinical stage of development, sixty-three are being tested in clinical studies on humans and fifteen reached phase three of development and are being tested for efficacy on a large scale [8]. Moreover, at present, the European Medicines Agency (EMA) is considering four COVID-19 vaccines through the “rolling review” procedure, a regulatory tool to speed up the assessment of medicines or vaccines during a public health emergency and consisting of reviewing data as they become available, before the submission of the formal application for marketing authorisation [4]. In particular, two candidate mRNA-based vaccines include BNT162b2 and mRNA-1273, and two non-replicating viral vector vaccines include ChAdOx1 nCoV-19 and Ad26.COV2.S [7,8]. Currently, BNT162b2 and mRNA-1273 vaccines have been approved in several countries. On 2 December 2020, the United Kingdom granted emergency authorization to the BNT162b2 vaccine, thus being the first Western country to give an approval to a coronavirus vaccine [9], followed some days later by the Food and Drug Administration (FDA) in the US [10]. Shortly after, on 21 December 2020, the BNT162b2 vaccine was authorised across the EU following EMA’s recommendation for a conditional marketing authorization [11]. As regards the mRNA-1273 vaccine, it received emergency use authorization by the FDA on 18 December 2020 [12]. Considering this candidate and the European context, the assessment by the EMA of the marketing authorization application is ongoing and at the beginning of January 2021 a meeting will take place to possibly conclude it [13].

To emphasize the importance of the availability of the COVID-19 vaccine, the European Commission has diversified vaccine portfolios and concluded six different contracts with pharmaceutical companies. These vaccine candidates are in an advanced phase of clinical trials and once the vaccines have been proven to be safe and effective, they will be quickly spread. Furthermore, the different types of development of vaccine platforms will ensure the possibility of different strategies [14,15,16]. While the development and availability of vaccines can be accelerated, standards for vaccine quality, safety and efficacy remains a fundamental prerequisite for marketing authorisation [4]. In addition, as highlighted by the technical report of the European Centre for Disease Prevention and Control (ECDC, 26 October 2020), a sustained monitoring programme based on post-marketing studies on the impact, effectiveness and safety of deployed vaccines, as well as routine reporting of adverse events following immunisation, will be crucial to inform policy decisions and to provide timely evidence [17].

Several official documents provide indications on potential priority groups to consider when designing and implementing vaccination programmes [4,17] and a survey carried out by the ECDC on vaccination strategies and vaccine deployment plans in the EU/EEA and UK highlighted that, as of 30 November 2020, nine countries have already published interim recommendations for priority groups [18]. The need for a flexible approach, sensitive to local conditions is essential; strategies should adapt to emerging evidence, epidemiological situations, vaccine delivery system capacities and the availability of vaccines themselves, among others. Surely, the starting point for planning is the objective of the vaccination programme: should vaccination prevent death and alleviate disease burden, or should it counteract the spread of the virus? To answer this question both the target population and the type of vaccine being used, considering the different endpoints that different types of vaccines can have, need to be taken into account. If the primary aim is to reduce COVID-19-related disease and deaths, while at the same time reducing the strain on healthcare systems, vulnerable people at higher risk to severe or fatal outcomes because of age or underlying conditions should be prioritized (older age, hypertension, diabetes, cardiovascular disease, chronic respiratory disease, chronic kidney disease and obesity, etc.), together with healthcare workers and essential workers with a significant risk of exposure. As long as the availability of COVID-19 vaccines is limited, categories at risk of severe disease should be privileged even among healthcare workers and essential workers with a significant risk of exposure.

Later on, assuming greater availability of COVID-19 vaccines, vaccination strategies could adapt and also include younger individuals in order to address asymptomatic transmission, that largely contributes to the spread of SARS-CoV-2 and that is most common in younger age segments, therefore limiting the circulation of the virus and indirectly providing protection to the most vulnerable individuals. However, in this second scenario it must be proven that vaccines prevent not only symptomatic severe disease but also infection and its transmission. Moreover, the safety profile should be highly rigorous because of the low risk of young people in case of infection and their longer life expectancy.

COVID-19 vaccination strategies should be carefully planned and should account for limited availability, especially early after marketing authorization, posing a great challenge in terms of both prioritization and organization. Of capital importance, the issue to be addressed is COVID-19 vaccine distribution and allocation, which is directly related to ethical considerations regarding equitable worldwide access once the vaccines will be available [19]. Defining risk groups to be taken into account for vaccination access and their prioritization is of great importance, in addition to keeping in mind that socio-economic conditions highly contribute to the burden of this disease; indeed, COVID-19 could be defined as a syndemic, rather than a pandemic, accounting for social and environmental factors that could have a critical role in promoting and enhancing the disease [20].

At present, there are no definite estimates on the number of doses that will be potentially available and on the timing of supply. In order to design and implement a feasible and efficient vaccination programme, delivery aspects need to be properly taken into account and could include type of settings different from the usual ones, according to the target population [17]. Vaccination services should be easily accessible and should also be adequately equipped with sufficient resources in terms of personal protective equipment (PPE) [4]. Furthermore, vaccine storage issues (such as considerations regarding extremely low temperatures some vaccines must be kept at) and the number of required doses per person, thus, the possible need for follow-up will play a fundamental role in the logistics; dedicated personnel should be adequately trained and prepared for a possibly high load vaccination campaign [17]. On the other side, nonetheless, targeted, tailored and transparent communication campaigns are critical in building trust and ensuring that the adequate vaccination coverage is reached, counteracting misinformation and vaccine hesitancy.

The great attention on the COVID-19 vaccine should not distract the focus from other vaccinations. For example, an adequate coverage against flu is crucial to limit the spread of this respiratory virus, to reduce the impact on healthcare systems and to facilitate differential diagnosis with COVID-19 in specific segments of the population at higher risk of symptomatic and severe disease.

In conclusion, before the introduction of one or more COVID-19 vaccines, it is of high importance that policy makers, professional and scientific societies and civil society organizations are prepared with vaccine delivery infrastructure, assessing different scenarios and the impact of alternative vaccination strategies. Lessons learned from the H1N1 pandemic should also be considered. In synthesis, key components for successful policy and vaccination programmes include timely, flexible and evidence-based decisions, robust surveillance systems for COVID-19 disease and monitoring of adverse events following immunization, communication plans and equitable access to vaccination. The promise of the new COVID-19 vaccine is great, but there are further challenges yet to be overcome.
